# Exploring the Transformative Potential of Functionalized Mesoporous Silica in Enhancing Antioxidant Activity: A Comprehensive Review

**DOI:** 10.3390/antiox13080936

**Published:** 2024-08-01

**Authors:** Arif Budiman, Agus Rusdin, Yoga Windhu Wardhana, Lisa Efriani Puluhulawa, Faradila Ratu Cindana Mo’o, Nurain Thomas, Amirah Mohd Gazzali, Diah Lia Aulifa

**Affiliations:** 1 Department of Pharmaceutics and Pharmaceutical Technology, Faculty of Pharmacy, Universitas Padjadjaran, Jl. Raya Bandung-Sumedang Km. 21, Bandung 45363, Indonesia; agusrusdin@gmail.com (A.R.); y.w.wardhana@unpad.ac.id (Y.W.W.); 2 Department of Pharmacy, Faculty of Sport and Health, Universitas Negeri Gorontalo, Jl. Jenderal Sudirman No. 6, Gorontalo 96128, Indonesia; lisapuluhulawa@ung.ac.id (L.E.P.); faradilaratu@ung.ac.id (F.R.C.M.); nurain.thomas@ung.ac.id (N.T.); 3 Department Pharmaceutical Technology, School of Pharmaceutical Sciences, Universiti Sains Malaysia, Penang 11800, P. Penang, Malaysia; amirahmg@usm.my; 4 Department of Pharmaceutical Analysis and Medicinal Chemistry, Faculty of Pharmacy, Universitas Padjadjaran, Bandung 45363, Indonesia; diah.lia@unpad.ac.id

**Keywords:** functionalized mesoporous silica, antioxidant, encapsulation, drug delivery

## Abstract

Antioxidants are essential for reducing oxidative stress, protecting cells from damage, and supporting overall well-being. Functionalized mesoporous silica materials have garnered interest due to their flexible uses in diverse domains, such as drug delivery systems. This review aims to thoroughly examine and evaluate the progress made in utilizing functionalized mesoporous silica materials as a possible approach to enhancing antioxidant activity. The authors performed a thorough search of reliable databases, including Scopus, PubMed, Google Scholar, and Clarivate Web of Science, using precise keywords linked to functionalized mesoporous silica nanoparticles and antioxidants. The identified journals serve as the major framework for the main discussion in this study. Functionalized mesoporous silica nanoparticles have been reported to greatly enhance antioxidant activity by allowing for an increased loading capacity, controlled release behavior, the targeting of specific drugs, improved biocompatibility and safety, and enhanced penetration. The results emphasize the significant capacity of functionalized mesoporous silica (FSM) to bring about profound changes in a wide range of applications. FSM materials can be designed as versatile nanocarriers, integrating intrinsic antioxidant capabilities and augmenting the efficacy of current drugs, offering substantial progress in antioxidant therapies and drug delivery systems, as well as enhanced substance properties in the pharmaceutical field. Functionalized mesoporous silica materials are a highly effective method for enhancing antioxidant activity. They provide new opportunities for the advancement of cutting-edge treatments and materials in the field of antioxidant research. The significant potential of FSM materials to change drug delivery methods and improve substance properties highlights their crucial role in future breakthroughs in the pharmaceutical field and antioxidant applications.

## 1. Introduction

Antioxidants are vital for maintaining cellular health and preventing various diseases, and they acts as the first line of defense against oxidative stress. Their ability to neutralize free radicals makes them indispensable components in the pursuit of overall well-being [1,2]. As our understanding of antioxidants deepens, researchers seek novel approaches to enhance their effectiveness. One such avenue is the integration of antioxidants with mesoporous silica materials, a concept that has gained momentum in recent years [3,4,5].

Mesoporous silica, characterized by its ordered and tunable pore structures, offers a versatile platform for encapsulating and delivering bioactive compounds [6]. Functionalized mesoporous silica has functional groups on its silica matrix surface, which can enhance its compatibility with specific substances, enabling tailored interactions [7,8,9]. This section explores the structural attributes of mesoporous silica, highlighting its potential for improving antioxidant performances. The synergy between functionalized mesoporous silica and antioxidants presents an exciting prospect for advancing antioxidant delivery systems.

Functionalized mesoporous silica materials hold immense promise in augmenting the antioxidant activity of compounds through various mechanisms of action [10]. This section delves into the reported strategies employed to enhance antioxidant efficacy, including improvements in loading capacity, controlled release, improved stability, enhanced biocompatibility, reduced doses and reduced potential side effects, increased protection against environmental factors, and providing a targeted drug delivery system [11,12]. By comprehensively analyzing the outcomes of individual studies, this review aims to uncover the intricacies of the interactions between functionalized mesoporous silica and antioxidant compounds.

While the literature boasts numerous original research articles on the use of functionalized mesoporous silica nanoparticles (MSNs) for antioxidant delivery, there is a notable gap in comprehensive reviews on this topic. The existing reviews related to MSNs in pharmacology, toxicology, and pharmaceutics are typically limited to discussing “Advances in functionalized mesoporous silica nanoparticles for tumor-targeted drug delivery and theranostics” [13] and “Functionalized mesoporous silica particles for application in drug delivery systems” [14]. However, there is no review specifically dedicated to the use of mesoporous silica nanoparticles for antioxidant compounds. Despite the wealth of research on the development of MSNs for antioxidant compounds, a consolidated review that systematically summarizes and analyzes these advancements has yet to be published. This review addresses this critical gap by offering a thorough and in-depth exploration of the success of functionalized mesoporous silica in enhancing antioxidant activity. In light of the aforementioned considerations, the primary aim of this review is to critically evaluate and synthesize the existing research on the utilization of functionalized mesoporous silica materials to improve the antioxidant activity of compounds. By examining key trends, challenges, and potential avenues for future research, the authors aim to contribute to a deeper understanding of the synergistic relationship between functionalized mesoporous silica and antioxidant compounds, fostering advancements in this burgeoning field.

## 2. Methodology

The methodology for this review involved a meticulous literature search conducted on reputable databases, including Scopus, PubMed, Google Scholar, and Clarivate Analytics Web of Science, using the specific keywords ‘functionalized’, ‘silica’, ‘mesoporous’, and ‘antioxidant’. The search parameters were set to include articles with the keywords in their title, abstract, or keywords. To ensure the incorporation of the latest advancements, only articles published since 2020 were considered for the main discussion. The focus was exclusively on original articles, filtering for studies published in journals specializing in silica mesoporous research and emphasizing functionalization with a specific examination of antioxidant activity. Following the retrieval of relevant articles, a systematic data extraction and organization process was employed, scrutinizing each article for key information such as the experimental methodologies used, outcomes, and conclusions. The data were then categorized thematically to facilitate a structured presentation in the main discussion of the review. A quality assessment was conducted to evaluate the methodology, experimental design, and overall rigor of each study, ensuring the inclusion of high-quality research in the synthesis of the current state of knowledge. This comprehensive methodology aims to provide a nuanced and in-depth analysis of the literature, offering valuable insights into the potential of functionalized mesoporous silica materials in enhancing the activity of antioxidant compounds.

## 3. Functionalized Mesoporous Silica: An Overview

### 3.1. Definition and Characteristics of Mesoporous Silica

Mesoporous silica nanoparticles (MSNs) are a unique class of materials with a sponge-like structure [15,16]. They consist of silicon dioxide (SiO_2_), the same chemical compound as sand, but at a much smaller size, typically less than 1 micrometer in diameter [15]. What sets them apart is their intricate network of pores ranging from 2 to 50 nanometers in size. This falls within the mesoporous range according to the IUPAC classification (International Union of Pure and Applied Chemistry) [17].

**Figure 1:**
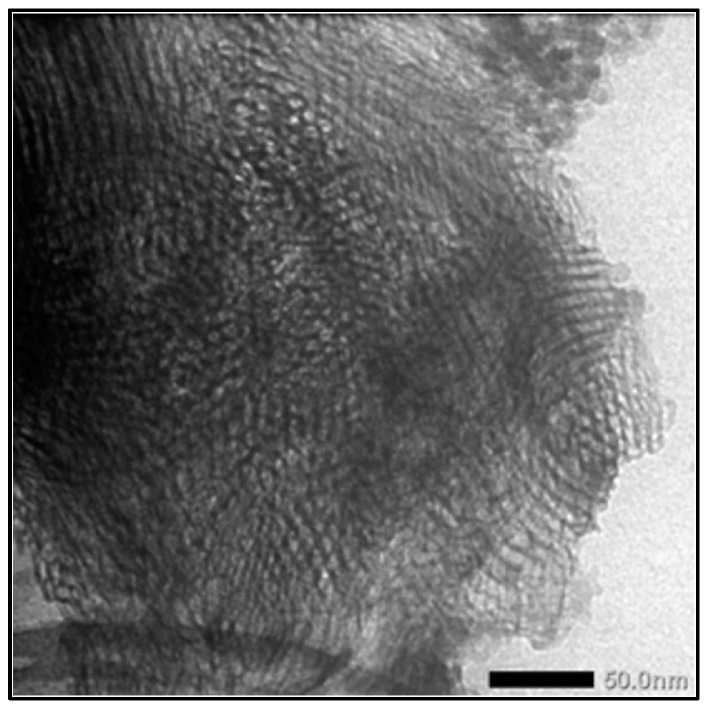
TEM image of MS nanoparticles. Adapted from data presented originally in Ref. [12].

Mesoporous silica nanoparticles (MSNs) exhibit several key characteristics that render them remarkably versatile. Firstly, their high surface area, akin to one American football field per gram, facilitates the extensive adsorption and encapsulation of various molecules [18]. Secondly, the tunable pore size of MSNs enables precise control over the size of the molecules that are loaded and released, distinguishing them from conventional silica (Figure 1). Thirdly, their biocompatibility makes them attractive for biomedical applications, such as drug delivery, as they pose a minimal threat to living organisms [19,20]. Mesoporous silica nanoparticles (MSNs) can be broadly categorized based on their pore size, with each category exhibiting distinct properties and functionalities (Table 1).

Additionally, the facile surface modification of MSNs with different functional groups permits targeted interactions and controlled release properties. These combined attributes have positioned MSNs as highly appealing for diverse applications including catalysis, environmental remediation, use in sensors, and notably, the drug delivery of antioxidant compounds. By encapsulating antioxidants within the pores of MSNss, scientists aim to enhance their stability, regulate their release, and target specific sites within the body, making MSNs pivotal in advancing therapeutic interventions [21,22,23].

### 3.2. Functionalized Mesoporous Silica Nanoparticles

Mesoporous silica nanoparticles (MSNs) are being widely used in the field of biomedicine due to their distinct characteristics and adaptability. Their unique attributes, including their excellent biocompatibility, adjustable pore size and volume, and expansive surface area, make them highly suitable for applications in drug delivery, imaging, and biosensing. Nevertheless, in order to fully unleash their capabilities, MSNs are frequently in want of further functionality. This is accomplished through a procedure called functionalization, in which the surface chemistry of MSNs is carefully altered to incorporate particular functions [24].

The functionalization of mesoporous silica nanoparticles (MSNs) can be accomplished using a variety of well-established processes. By grafting organic molecules onto their surface, it becomes possible to include targeted ligands, responsive polymers, or imaging agents. Targeting ligands, like antibodies or peptides, are capable of specifically recognizing receptors on miserable cells. The targeted delivery technique improves the effectiveness of treatment by concentrating the drugs at the intended area while minimizing exposure to healthy tissues and reducing negative effects. Conversely, stimuli-responsive polymers can be engineered to release a substance in reaction to particular environmental signals, such as alterations in the pH or temperature. The implementation of this regulated discharge mechanism presents notable benefits compared to conventional methods of administering medication, potentially enhancing the effectiveness of treatment and diminishing the overall toxicity to the body [15,25].

The incorporation of organic substances enhances the capabilities of MSNs. For example, through the incorporation of magnetic nanoparticles, it becomes possible to utilize external magnetic fields to direct the nanoparticles towards specific tissues, hence improving targeted delivery. Alternatively, the incorporation of fluorescent or luminous materials allows for the utilization of MSNs in in vivo imaging applications. By monitoring the position and motion of these modified nanoparticles inside the body, scientists can acquire crucial knowledge about biological mechanisms and assess the effectiveness of treatments [26,27].

Functionalized mesoporous silica nanoparticles (MSNs) demonstrate great potential for biosensing applications, in addition to their use in drug delivery and imaging. Researchers can develop very precise and sensitive sensors for detecting different biomolecules, including disease biomarkers, by binding biorecognition molecules like enzymes or antibodies on the surfaces of MSNs. These sensors have the potential to be utilized for early detection, the monitoring of diseases, and the development of individualized treatment strategies [28].

Functionalized MSNs represent a powerful platform for diverse biomedical applications. Their tailored functionalities enable targeted drug delivery, enhanced imaging capabilities, and the development of novel biosensors. As research in this field continues to advance, functionalized MSNs are poised to revolutionize various aspects of healthcare and disease management.

### 3.3. Techniques for Functionalization

The ability to manipulate the surface chemistry of MSNs is crucial for unlocking their full potential in various applications [29]. Functionalization refers to the introduction of desired functional groups onto the silica surface, either during the synthesis process (co-condensation) or after the initial formation (post-synthesis grafting), or using other methods such as click chemistry and electrostatic assembly. These functional groups can endow MSNs with specific properties, allowing them to interact with target molecules, respond to external stimuli, or control the release of encapsulated cargo [30,31].

#### 3.3.1. Co-Condensation

Co-condensation is a widely employed technique for directly incorporating functional molecules within the silica framework during MSN synthesis. This approach offers several advantages over post-synthesis grafting methods. Here, organosilanes, molecules containing an organic group bonded to a silicon atom and three alkoxy groups (e.g., methoxy, ethoxy) [32,33], play a pivotal role. During co-condensation, the alkoxy groups of the organosilane precursors co-react with the silica precursor (typically tetraethylorthosilicate, TEOS) to form Si-O-Si linkages. This process covalently tethers the desired organic functionalities to the MSN framework [24].

Co-condensation has unique benefits. In the beginning, it enables a uniform spread of functional groups across the MSN structure, guaranteeing consistent performance. Furthermore, the quantity of the organosilane precursor used enables accurate regulation of the density of functional groups on the resulting MSNs. Researchers can customize the surface qualities for specific applications using this control method. In addition, co-condensation has the potential to offer a wider range of functionalities compared to post-synthesis grafting. This is because the organosilane precursors can be easily constructed with various organic moieties [34].

However, co-condensation also presents some limitations. Careful selection of the organosilane precursors is crucial to ensure compatibility with the synthesis conditions. The size and functionality of the organosilane molecule can significantly impact the final pore structure and overall properties of the MSNs [35,36]. Incompatible precursors can disrupt the self-assembly process, leading to the formation of defective MSNs with compromised pore characteristics. Therefore, optimizing the co-condensation process often requires careful consideration of the chosen organosilane precursors and their interaction with the silica source under the chosen synthesis conditions.

Co-condensation is a highly effective method for producing functionalized MSNs. The advantages of this method lie in its ability to distribute functional groups evenly, adjust the MSNs’ density with precision, and incorporate a wider range of functional groups. This makes it highly valuable for researchers. However, meticulousness in the choosing of organosilane precursors and fine-tuning of the synthesis conditions are crucial for achieving successful application.

#### 3.3.2. Post-Synthesis Grafting

Post-synthesis grafting offers an alternative approach to introducing functionalities onto pre-formed mesoporous silica nanoparticles (MSNs). This method typically follows the template removal process, often conducted at room temperature for around 6 h [37]. It relies on the abundant silanol groups (Si-OH) present on the MSN surface, which act as reactive sites for attachment. The grafting process involves the reaction of these silanol groups with suitable silanes or coupling agents. These coupling agents bear two key functionalities: one that reacts with the silanols, forming a covalent bond with the MSN framework, and another reactive group that can subsequently bind to the desired functional molecule [38].

Post-synthesis grafting offers numerous benefits. Firstly, it demonstrates a wider range of usefulness. Contrary to co-condensation, which may be restricted by the compatibility of organosilanes with the synthesis conditions, post-grafting can be used on a broader spectrum of MSNs, produced using different methods. This adaptability enables the incorporation of functionalities that may not be suitable for the elevated temperatures or reaction conditions used in co-condensation. Furthermore, post-grafting provides enhanced manipulation of the surface chemistry of MSNs for characterization after synthesis. Researchers can customize the functionalization of the MSN characteristics to meet specific application requirements once they have fully defined them [39].

Nevertheless, there are constraints linked to post-synthesis grafting. An issue arises from the possibility of an uneven distribution of functional groups within the MSN structure. Grafting is more likely to occur on the outside surface because it is more easily accessible than the inside pores. This might result in a varied distribution of functionalities, which has the potential to impact the performance of the MSNs. Another issue to consider is the potential impact of the grafting process on the pore size and volume of the MSNs. The overall accessibility of the pores can be reduced due to pore obstruction or shrinkage, which depends on the size and bulkiness of the grafted molecules [40,41].

The decision between co-condensation and post-synthesis grafting depends on the desired functionality and the specific features of the MSN being targeted. Gaining a comprehensive understanding of the benefits and constraints of both strategies enables researchers to choose the most appropriate method for achieving precise control over the surface chemistry of MSNs. Controlling this factor is essential for the development of these adaptable materials for specific purposes in drug administration, imaging, and other fields of biomedical investigation [39,40,41].

#### 3.3.3. Click Chemistry

Click chemistry has become a potent method for precisely adding functionality to pre-modified mesoporous silica nanoparticles (MSNs). This methodology utilizes very precise and efficient interactions between complimentary functional groups, providing significant advantages compared to conventional conjugation approaches. Click reactions are well-known for their great efficiency in producing desired products, their gentle reaction conditions that prevent damage to delicate biomolecules, and their ability to reduce the formation of unwanted byproducts [42,43]. These characteristics translate to improved control over the functionalization process and the final properties of the MSNs.

Specific click reactions, such as the highly popular azide-alkyne cycloaddition (CuAAC) or thiol-ene coupling, serve as the cornerstone of this technique. These reactions involve the precise coupling of pre-installed functional groups on the MSN surface with complementary moieties present on the desired functional molecules [44]. For instance, azide-modified MSNs can be readily conjugated with alkyne-tagged biomolecules or targeting ligands via CuAAC, resulting in the formation of a stable triazole linkage. Similarly, thiol groups on the MSN surface can be efficiently coupled with alkene-functionalized molecules through thiol-ene click chemistry.

The versatility of click chemistry stems from the extensive range of clickable functional groups that can be included into both the MSN and the target molecule. Researchers can customize the surface features of MSNs with exceptional accuracy, which allows them to create nanocarriers that are highly functionalized for various biomedical uses. Click chemistry provides a more precise and effective way of functionalizing MSNs compared to standard conjugation approaches, which frequently need severe reaction conditions or lack specificity [42,44].

Overall, click chemistry serves as a beneficial method for the creation of precisely functionalized MSNs. This technique is very appealing to researchers aiming to develop improved nanomaterials for targeted drug delivery, biosensing, and other biomedical applications due to its advantages in terms of high yields, gentle reaction conditions, and the potential to add a wide range of functions.

#### 3.3.4. Electrostatic Assembly

Electrostatic assembly provides a direct method to modify MSNs by utilizing the attraction forces between objects with opposite charges. This approach depends on the surface charge of the MSNs, which may be customized during synthesis to be either positively or negatively charged. Functional molecules, such as proteins or nucleic acids, can be attached to the surface of MSNs through electrostatic adsorption, provided that they have a charge that complements the surface charge of the MSNs [45]. This approach offers several advantages, particularly for the incorporation of biomolecules that might be sensitive to harsher functionalization methods.

The primary benefit of electrostatic assembly is its inherent simplicity. The procedure entails a limited number of steps and often takes place under calm circumstances, rendering it appropriate for biomolecules that may be susceptible to deterioration at elevated temperatures or in the presence of potent chemicals. The mild nature of this process is especially helpful for the encapsulation of proteins, enzymes, and nucleic acids, as it allows them to maintain their bioactivity to a larger degree than other techniques of functionalization [46].

Electrostatic assembly is highly advantageous for the targeted delivery of drugs and biosensing applications. By attaching cationic targeting ligands onto the surfaces of anionic MSNs, researchers can produce nanoparticles that selectively adhere to specific cells or tissues that possess anionic receptors. This focused strategy improves the effectiveness of therapy and reduces unintended effects in the field of drug delivery [45]. Similarly, in biosensing, MSNs functionalized with complementary-charged biorecognition molecules can be used to selectively detect specific biomolecules through electrostatic interactions.

Nevertheless, there are constraints to take into account when it comes to electrostatic assembly. The main difficulty arises from the possibility of non-covalent interactions occurring between the surface of the MSN and the functional molecule. These contacts may have a relatively low strength, which could result in the gradual release of the payload that is being transported, especially under normal physiological environments. Furthermore, the management of the surface charge density of MSNs and of the quantity of functional molecules they carry can be difficult, potentially impacting the overall effectiveness of the modified MSNs [47].

Although electrostatic assembly has limits, it is yet a valuable approach for functionalizing MSNs, especially for applications involving biomolecules that need to be handled delicately. Through the meticulous analysis of charge interactions and possible leakage, researchers can utilize this method to develop functionalized MSNs for precise drug delivery, biosensing, and various biological applications. 

### 3.4. Brief Review of Previous Studies Involving Mesoporous Silica in Pharmaceutical Applications

Mesoporous silica nanoparticles (MSNs) have emerged as a powerful platform for various pharmaceutical applications due to their unique properties.

#### 3.4.1. Drug Delivery

Mesoporous silica nanoparticles (MSNs) are highly promising for encapsulating diverse therapeutic agents due to their high surface area and tunable pore size [48]. Loading drugs within MSN pores offers several advantages, including an enhanced solubility for poorly soluble drugs, targeted delivery through surface ligand attachment to specific cells or tissues, and controlled release governed by factors such as the pore size, surface functionalization, and external stimuli [49,50]. This controlled release enables optimized drug delivery with sustained or triggered release profiles. Previous studies have extensively investigated MSNs for delivering various drugs, including anticancer agents, antibiotics, and gene therapy vectors [51]. For example, research has demonstrated that MSNs loaded with doxorubicin, a chemotherapy drug, exhibited improved antitumor activity compared to the free drug. Furthermore, functionalizing MSNs with antibodies has shown efficacy in targeting specific cancer cells, thereby enhancing drug delivery while minimizing systemic exposure. Such findings underscore the potential of MSNs as versatile platforms for advanced drug delivery systems [52,53].

#### 3.4.2. Bioimaging

Mesoporous silica nanoparticles (MSNs) offer a versatile platform for functionalization with fluorescent dyes or other imaging agents, enabling their visualization within the body via techniques such as fluorescence imaging or magnetic resonance imaging (MRI) [54,55]. This capability holds significant value for tracking drug delivery, as incorporating imaging moieties into drug-loaded MSNs allows researchers to monitor their biodistribution and release in real-time [56]. Moreover, MSNs functionalized with specific targeting molecules can facilitate the diagnostic imaging of diseased tissues or specific biomarkers. Previous studies have showcased the utility of MSNs for the in vivo imaging of tumors and the monitoring of stem cell migration, highlighting their potential for non-invasive diagnostics and theranostic applications, which integrate therapy and diagnosis seamlessly [54,57,58]. Further studies have emphasized the efficacy of mesoporous silica nanoparticles (MSNs) in the fields of bioimaging and targeted cancer therapy. Shirani et al., in 2023 created a modified version of MSN-NH2 by incorporating gemcitabine (GEM) and coating it with carbon dots (CDs) to improve theimaging and targeting capabilities. The nanocarrier FA/CDs/MSN-NH2@GEM, modified with folic acid, exhibited an excellent targeting ability and cytotoxicity against HeLa and K562 cancer cells. This was confirmed by the use of fluorescence microscopy and flow cytometry [59]. Kumar et al., in 2023 developed mesoporous silica-encapsulated iron oxide (IO) nanocomposites for the purpose of delivering doxorubicin (DOX) in a magnetically guided manner. The nanocomposites exhibited a regulated drug release that was based on pH, demonstrated considerable toxicity to cancer cells when exposed to magnetic fields, and did not exhibit toxicity towards healthy cells. Both findings confirm the effectiveness of MSNs in improving medication delivery, targeted specificity, and bioimaging, which makes them viable tools for the treatment of cancer [60].

#### 3.4.3. Biosensing

The ability of MSNs to interact with biomolecules makes them suitable for developing biosensors for disease detection [61,62]. By incorporating enzymes, antibodies, or other biorecognition elements onto the MSN surface, researchers can create sensors that can selectively bind to specific analytes (target molecules) [62]. This binding event can then be transduced into a measurable signal (e.g., fluorescence change) for detection. Previous studies have explored the use of MSNs for the detection of various biomarkers, including glucose and cancer antigens. These sensors offer advantages like high sensitivity, good bio-compatibility, and the potential for point-of-care diagnostics [63]. Another advancement highlights the effectiveness of mesoporous silica nanoparticles (MSNs) in biosensing applications. Aghayan et al., in 2023, synthesized copper-functionalized mesoporous silica nanoparticles (Cu-MSN) using a sol-gel method. These nanoparticles demonstrated enhanced peroxidase-like activity in comparison to other types of nanoparticles. The Cu-MSNs showed high efficiency in detecting H_2_O_2_ and glutathione (GSH), with detection limits of 0.2 μM and 0.0126 μM, respectively. The validation of these results was performed using human serum samples [64]. In a similar manner, Xing et al., in 2022 produced mesoporous silica nanoparticles (MSNs) that were modified with fluorescent molecules obtained from benzimidazole (DHBM). This synthesis resulted in a detection limit of 7.69 × 10^−8^ M for Cu^2+^ in several types of materials, such as bovine serum and live cells. Both results emphasize the exceptional sensitivity, specificity, and practical use of MSNs in biosensing, opening up possibilities for groundbreaking biological applications [65].

## 4. Antioxidant Activity of Active Pharmaceutical Ingredients (API): Fundamental Concepts

### 4.1. Importance of Antioxidants in Pharmaceuticals

Counter-acting free radical damage is a critical aspect of pharmaceutical research, given the detrimental impact of these highly reactive molecules on cellular and tissue integrity [66]. Free radicals, characterized by unpaired electrons, indiscriminately damage essential biomolecules such as proteins, lipids, and DNA, contributing to the pathogenesis of various chronic diseases including cancer, cardiovascular diseases, and neurodegenerative disorders [67,68]. Antioxidants play a pivotal role in combating free radical-induced damage by acting as scavengers, neutralizing the free radicals by donating electrons and thereby preventing further molecular harm. In pharmaceuticals, antioxidants offer dual benefits [69]. Firstly, they protect drugs from degradation caused by oxidation, preserving their structural integrity and therapeutic efficacy within formulations. Secondly, antioxidants can mitigate oxidative stress associated with certain disease states, potentially improving patient outcomes when incorporated into medications or therapeutic regimens. This multifaceted role underscores the importance of antioxidants in pharmaceutical formulations aimed at combating free radical damage and promoting health and wellness [70,71]

Antioxidants play a crucial role in mitigating oxidative stress by interacting with free radicals through electron transfer mechanisms. A representative chemical reaction involving an antioxidant molecule (AH) and a free radical (R*) can be depicted as follows:

AH + R* → A* + RH

AH: Antioxidant species;
R*: Free radical species;
A*: Signifies the radical form of the antioxidant subsequent to electron donation
RH: Stable product subsequent to antioxidant-mediated free radical neutralization.

This process exemplifies the antioxidant’s ability to scavenge free radicals by donating electrons, thereby quenching their reactivity and contributing to cellular homeostasis and defense against oxidative damage. Such interactions are pivotal in understanding the biochemical roles of antioxidants in biological systems.

Preserving the stability of pharmaceutical formulations is paramount to ensuring the efficacy and safety of medicinal products, particularly in light of their susceptibility to environmental factors such as light, oxygen, heat, and moisture. These factors can catalyze oxidation reactions, leading to the degradation of active ingredients and the formation of potentially harmful byproducts [72,73]. Antioxidants play a crucial role in formulation stability by acting as stabilizers, effectively mitigating these deleterious effects through several mechanisms. Firstly, antioxidants can chelate metal ions present in formulations, thereby preventing them from catalyzing oxidation reactions and accelerating drug degradation. Additionally, antioxidants are capable of quenching singlet oxygen, a highly reactive form of oxygen known to cause oxidative damage to drugs and other molecules. By leveraging these mechanisms, antioxidants serve as indispensable components in pharmaceutical formulations, safeguarding the integrity and potency of medicinal products against environmental stresses and ensuring their therapeutic effectiveness and safety for patient use.

Enhancing drug delivery represents a significant application of antioxidants in pharmaceutical research, with potential benefits in terms of improving drug solubility and efficacy, as well as mitigating inflammation-related complications [74,75]. Firstly, antioxidants with amphiphilic properties, featuring both water-soluble and fat-soluble regions, hold promise for enhancing the solubility of poorly soluble drugs. By aiding in the dispersion of hydrophobic compounds, these antioxidants facilitate their absorption and distribution within the body, thereby improving their bioavailability and therapeutic effectiveness. Additionally, antioxidants exhibit anti-inflammatory properties, which are particularly relevant in conditions characterized by heightened oxidative stress and inflammation. By reducing inflammation, antioxidants can complement the actions of medications used to treat inflammatory conditions, potentially enhancing their efficacy and reducing associated complications. Leveraging these capabilities, antioxidants offer valuable opportunities for optimizing drug delivery strategies, ultimately contributing to improved therapeutic outcomes and patient well-being.

### 4.2. Types of Antioxidants Used in Pharmaceuticals

Antioxidants, whether synthetic or natural, play indispensable roles in pharmaceutical formulations by effectively combating oxidative degradation and preserving the stability and efficacy of active ingredients (Table 2). Synthetic antioxidants such as butylated hydroxyanisole (BHA) and butylated hydroxytoluene (BHT) are widely utilized for their robust antioxidative properties, effectively preventing drug degradation within formulations and ensuring product integrity. Conversely, natural antioxidants derived from vitamins (such as C and E), plant phenolics (including curcumin and resveratrol), and other bioactive compounds offer distinct advantages beyond antioxidative activity. These natural antioxidants may possess additional therapeutic benefits, such as anti-inflammatory properties, or synergistically interact with therapeutic drugs, enhancing their efficacy. Through a comprehensive understanding of the diverse properties and mechanisms of action of both synthetic and natural antioxidants, pharmaceutical scientists can tailor formulations to optimize their stability, efficacy, and patient outcomes [76,77].

### 4.3. Physicochemical Properties

The efficacy of an active API as an antioxidant is intricately linked to its physical and chemical properties, which govern its interactions with free radicals and other molecules. Key properties which influence antioxidant activity include their redox potential, solubility, and pKa [78]. The redox potential dictates the ease with which a molecule can donate or accept electrons, with lower potentials indicating stronger antioxidant activity as the molecules readily donate electrons to neutralize free radicals [79]. Solubility is crucial for the API to be available for interaction with free radicals; poorly soluble APIs may exhibit limited antioxidant activity in vivo due to restricted bioavailability. Furthermore, the acid dissociation constant (pKa) can influence antioxidant activity by affecting the ionization state of the API in different pH environments, with specific ionization states potentially favoring free radical scavenging [80]. Understanding these properties is essential for the designing of APIs with optimal antioxidant efficacy, facilitating the development of pharmaceutical formulations aimed at combating oxidative stress-related disorders, and enhancing therapeutic outcomes.

### 4.4. Optimizing Antioxidant Activity of APIs

Comprehending the factors which influence the antioxidant activity of active APIs empowers researchers to devise strategies for optimizing their efficacy in combating oxidative stress-related disorders. Structural modifications represent a key approach, whereby chemical alterations can be employed to introduce functional groups or modify the APIs’ structure to enhance their electron-donating ability and free radical scavenging potential. Additionally, the formulation design plays a crucial role, as formulating APIs with suitable excipients can improve their solubility and bioavailability, thus augmenting their in vivo antioxidant activity. Furthermore, combination therapy emerges as a promising strategy, as synergistic interactions between APIs with complementary antioxidant properties may yield a more potent therapeutic approach. By leveraging these strategies, researchers can advance the development of pharmaceutical interventions aimed at mitigating oxidative stress and improving patient outcomes in various pathological conditions [81,82].

### 4.5. Antioxidant Effectiveness IC50 Values

The IC50 value, which represents the concentration at which antioxidants effectively scavenge free radicals in an in vitro assay method, is a fundamental parameter for assessing their efficacy. It denotes the level of antioxidant concentration needed to suppress 50% of the activity of free radicals. Smaller IC50 values indicate a stronger ability to counteract free radicals, resulting in increased antioxidant efficacy (Table 3) [83].

The IC50 values can be affected by the particular assay method used. Various assays employ different sources of free radicals and reaction conditions, which may potentially affect the observed antioxidant activity. Therefore, making direct comparisons between IC50 values derived from different tests may not be completely reliable.

Moreover, it is important to take into account the significance of IC50 values in relation to their applicability in in vitro and in vivo conditions. Although in vitro experiments provide a controlled environment to evaluate antioxidant activity, it might be difficult to extrapolate these results to in vivo effectiveness. The intricacies of the biological system, including processes such as metabolism, absorption, and bioavailability, can have a substantial impact on the true capability of an antioxidant within a live creature.

IC50 values offer vital information on the antioxidant capacity of a chemical. Nevertheless, they should not be exclusively depended upon as an absolute measure of effectiveness. To fully comprehend the subject, one must take into account the particular test employed, the potential constraints of in vitro models, and the presence of other routes through which the antioxidant may exert its advantageous impacts [83,84].

### 4.6. Current Challenges and Strategies to Maximize Antioxidant Activity of APIs

Despite the potential advantages of leveraging the antioxidant activity of APIs, several notable challenges impede their comprehensive utilization. Firstly, achieving a delicate balance between antioxidant activity and the primary therapeutic effect of an API is essential to ensure efficacy without compromising intended pharmacological actions [85]. Secondly, accurately predicting and measuring in vivo antioxidant activity faces challenges due to complexities inherent in biological systems, necessitating the development of sophisticated models and predictive methods [68]. Thirdly, formulating APIs with potent antioxidant properties faces hurdles such as compatibility issues with excipients, maintaining bioavailability, and achieving targeted delivery. Moreover, regulatory considerations regarding the safety and efficacy of APIs, including their antioxidant activity, present substantial hurdles in navigating approval processes. Finally, under-standing the intricate interplay of synergistic and antagonistic effects with other molecules in the body is crucial for optimizing antioxidant therapy while minimizing unintended consequences. Addressing these challenges requires interdisciplinary collaboration and innovative approaches to fully exploit the therapeutic potential of APIs with antioxidant activity.

### 4.7. Strategies to Overcome Challenges

Strategically overcoming the challenges associated with harnessing the antioxidant potential of APIs requires innovative approaches across multiple fronts. Firstly, leveraging computational modeling tools can facilitate the prediction of in vivo activity based on in vitro data and the API structure, aiding in the selection of APIs with optimal antioxidant benefits. Secondly, the development of targeted delivery systems, such as liposomes or nanoparticles, holds promise for enhancing the bioavailability and tissue-specific delivery of APIs, thereby maximizing their antioxidant activity at the site of action. Additionally, adopting combinatorial approaches by combining APIs with known antioxidant activities with other drugs or antioxidant molecules may yield synergistic benefits, amplifying the overall antioxidant potential and potentially improving therapeutic outcomes. Furthermore, the advancement of sophisticated in vivo models that better mimic human physiological conditions is essential for obtaining accurate data on the true antioxidant activity of APIs within the body. In conclusion, addressing these challenges through continued research efforts in the areas of computational modeling, targeted delivery systems, combinatorial approaches, and advanced in vivo models is paramount to unlocking the full therapeutic potential of APIs with potent antioxidant properties, paving the way for the development of more effective and multifaceted therapeutic strategies. This approach can be done by adding functional agents such as copper, cerium, gallic acid, irganox, and others. For more details, see Table 4 below.

## 5. Case Studies and Experimental Findings

### 5.1. Presentation of Key Findings Regarding the Impact of Functionalized Mesoporous Silica on API Antioxidant Activity

Functionalized mesoporous silica (FSM) has emerged as a revolutionary platform for enhancing the activity of various antioxidants (Table 4). This review explores key findings from numerous studies, highlighting the versatility of FSMs in improving antioxidant activity across diverse domains.

#### 5.1.1. Significantly Improve Loading Capacity

The approach of functionalizing mesoporous silica greatly increases their ability of maintaining and effectively encapsulating antioxidant medicines, resulting in enhanced therapeutic effects. This enhancement can be linked to numerous explanations, such as an increased surface area, enhanced drug–silica interactions, and improved stability of the loaded substances.

Functionalized mesoporous silica exhibits a greater capacity for loading compared to non-functionalized mesoporous silica, mainly because of the incorporation of functional groups that have a strong interaction with the utilized drug molecules. Kumari et al. (2021) found that, by modifying chitosan–silica nanohybrids with thioglycolic acid and functionalizing them with 3-(trimethoxysilyl)-1-propane thiol, they were able to achieve a quercetin encapsulation effectiveness of 92.38%. This exceptional efficiency is a result of the thiol groups augmenting the contact between the silica surface and quercetin molecules via hydrogen bonding and other non-covalent interactions [93].

In a similar manner, Kumari et al. (2023) showed that the combination of amine-modified MCM-41 with chitosan succinate resulted in an encapsulation efficiency of 87.24% for curcumin. The amine groups enhanced the electrostatic contacts and bonding of hydrogen with curcumin, resulting in a higher loading capacity. On the other hand, non-functionalized mesoporous silica generally shows less strong connections with drug molecules, leading to lesser amounts of drug that can be loaded [98].

Functionalizing mesoporous silica not only increases the amounts of pharmaceuticals that can be loaded, but also enhances the way the drugs are released and improves their stability. In their study, Zou et al. (2022) created hollow mesoporous manganese-doped silica nanoparticles (LHMMSNs) that were functionalized with lactoferrin. These nanoparticles were used to transport resveratrol, with a drug loading efficiency of 39.32 ± 2.46%. The application of a lactoferrin coating created a suitable and stable setting, thereby inhibiting premature drug release and degradation [95].

Xu et al. (2022) conducted a study that demonstrated the effectiveness of quaternary ammonium-functionalized mesoporous silica nanoparticles coated with chitosan–sodium tripolyphosphate (CS-TPP), achieving a betanin loading capacity of 21.66%. In comparison, non-functionalized silica only obtained a loading capacity of 2.95%. The presence of functional groups on the silica surface increased the electrostatic interactions with betanin, while the CS-TPP coating offered extra stability and safeguarded against degradation [96].

These examples demonstrate that functionalizing mesoporous silica greatly improves the ability to load antioxidant medicines on it by utilizing mechanisms such as increased surface area and pore volume. Functionalization can enhance the surface area and pore volume, hence increasing the number of sites available for drug adsorption. Enhanced drug–silica functional groups such as amines, thiols, and quaternary ammonium compounds interact more strongly with drug molecules through hydrogen bonding and electrostatic interactions. This leads to improved stability and release profiles. Functional coatings like chitosan and lactoferrin protect drugs from degradation and regulate the rate at which they are released, ensuring therapeutic levels are maintained for longer durations.

Functionalized mesoporous silica enhances the loading capacity and encapsulation efficiency of antioxidant medicines, resulting in improved therapeutic effects. This enhancement is propelled by the incorporation of functional groups that enhance the interactions with drug molecules and offer improved stability and regulated release. Subsequent investigations should investigate novel functionalization techniques and their impacts on different categories of medications in order to enhance the efficiency of drug delivery systems.

#### 5.1.2. Provide Controlled Release Behavior

Functionalized mesoporous silica enhances the release properties of antioxidant drugs in comparison to non-functionalized mesoporous silica. This improvement has been defined using the addition of functional groups that interact with the drug molecules, which results in a more regulated and prolonged release. These enhanced release profiles are influenced by different processes, including the pH sensitivity, interaction strength, and specific interactions with functional groups.

The process of functionalizing mesoporous silica enables the customization of drug release patterns, hence improving the effectiveness and stability of antioxidant medicines by ensuring regulated release. Luo et al. (2021) showed that mesoporous silica nanorods with thiol-functionalized surfaces, containing the antioxidant N-(1,3-dimethyl)butyl-N′-phenyl-p-phenylenediamine, exhibited a continuous release pattern. The implementation of this controlled release mechanism extended the duration of the antioxidant’s effectiveness, thereby enhancing the anti-aging capabilities of styrene-butadiene rubber [89]. Notably, the rubber did not exhibit any blooming even after undergoing 168 h of thermal oxidative aging at a temperature of 100 °C. The thiol groups present on the surface of silica are anticipated to establish more robust connections with the antioxidant, therefore impeding quick release and guaranteeing a gradual and persistent release.

In a similar vein, Kumari et al. (2021) demonstrated that chitosan–silica nanohybrids, which were modified with 3-(trimethoxysilyl)-1-propane thiol, had a release pattern that was sensitive to changes in pH. Specifically, a greater amount of the medication was released in acidic settings (pH 5.0) as opposed to neutral conditions (pH 7.4). The pH-dependent release of the encapsulated antioxidants is beneficial for specifically targeting acidic environments, such as certain malignant tissues. This enhances the therapeutic efficiency of the antioxidants [93].

Brezoiu et al. (2020) found that the presence of acidic functional groups attached to the silica pore walls had an impact on the release of polyphenolic extracts from functionalized MCM-41 mesoporous silica. These groups promoted the release of a greater quantity of phytochemicals in PBS, emphasizing the influence of the functional group chemistry in regulating drug release. Under physiological settings, acidic groups have the ability to undergo protonation, which leads to changes in the contacts between the drug and the silica matrix. As a result, the release rate of the medication is modified [90].

The utilization of amine-functionalized mesoporous silica also exhibits substantial enhancements in release patterns. Rashidi et al. (2022) discovered that amine-functionalized mesoporous silica nanoparticles (AP-MSNs) exhibited a prolonged release of 2-tert-butylhydroquinone (TBHQ) [106]. The presence of amine groups enhances the electrostatic and hydrogen-bond interactions with the antioxidant, resulting in a decelerated and regulated release.

Additionally, Sumithaa and colleagues (2023) found that the use of polydiacetylene (PDA)-coated amino-functionalized mesoporous silica nanoparticles (AMSNs) effectively controlled the release of anticancer medicines, preventing premature release and allowing for faster release in acidic conditions [105]. The π-conjugated PDA gatekeepers improve the uptake of cells and guarantee a gradual and uninterrupted release, which is essential for sustaining therapeutic medication levels for long durations.

Leccese et al. (2022) investigated the release characteristics of the photoactive orange carotenoid protein (OCP) from SBA-15 nanoparticles. The study revealed that certain circumstances can activate the release of OCP, showcasing the ability of functionalized mesoporous silica to react to environmental stimuli, such as light or temperature, hence enhancing the customization of the release profile [92].

This research emphasizes various fundamental mechanisms via which functionalized mesoporous silica might improve and regulate the release characteristics of antioxidant medicines. Drug–silica contacts are enhanced by the presence of functional groups such as thiols, amines, and acidic moieties. These groups form stronger binding contacts with drug molecules, leading to a more gradual and regulated release. Moreover, the pH sensitivity of functional groups, such as amines and acids, allows for precise release in particular settings, such as acidic tumor tissues or the fluctuating pH levels in the gastrointestinal tract. Furthermore, functionalized silica with stimuli-responsive release capabilities can be engineered to release pharmaceuticals in reaction to particular stimuli, such as changes in temperature or exposure to light, therefore enabling drug administration as needed. In addition, the implementation of polymers or coatings, such as polydiacetylene, can effectively inhibit premature drug release and guarantee an extended therapeutic impact.

To summarize, functionalized mesoporous silica offers a flexible platform for improving the release characteristics of antioxidant medications. By customizing the chemical properties of the surface and integrating systems that respond to inputs, these materials have the potential to greatly enhance the effectiveness and durability of enclosed pharmaceuticals. Further investigation should prioritize the exploration of novel methods for functionalization and their influence on various categories of antioxidants and drug delivery applications.

#### 5.1.3. Provide Targeted Drug Delivery System

Functionalized mesoporous silica greatly improves the targeted delivery and selectivity of antioxidant drugs in comparison to non-functionalized mesoporous silica. The enhancements are primarily due to the use of various functional groups and superficial modifications that facilitate precise interactions with disease locations, such as cancer cells, and the development of systems that respond to stimuli.

Functionalized mesoporous silica enables precise delivery by using many processes, including the pH sensitivity, redox responsiveness, and particular ligand–receptor interactions. These methods guarantee the targeted delivery of antioxidant medications to the affected cells, therefore improving the effectiveness of treatment while reducing potential negative effects.

Purikova et al. (2022) presented a study showcasing the development of mesoporous silica nanoparticles (MSNs) that have been modified with methylthiopropyl units. These units serve as switches that respond to reactive oxygen species (ROS). The MSNs were modified to contain cerium oxide nanoparticles, which were then released in an environment with high levels of reactive oxygen species (ROS). The released nanoparticles effectively removed over 80% of hydrogen peroxide (H_2_O_2_) over a span of 10 min. The presence of reactive oxygen species (ROS) controls this specific release system, which demonstrates how functionalization can generate a discerning and reactive drug delivery mechanism. This mechanism directly amplifies the antioxidant effects in conditions of oxidative stress, which are frequently linked to cancer and other disorders [97].

In their study, Fatemi et al. (2024) presented a versatile hollow mesoporous silica-based nanocarrier (HMSN) that can effectively carry irinotecan (IRT) specifically to colorectal cancer cells. The HMSNs were modified with cerium and iron oxides and covered with bacterial-derived exopolysaccharide (EPS). The cerium and iron ions worked together with the medication to produce reactive oxygen species (ROS) specifically in the acidic tumor microenvironment and lysosomes. The redox-responsive system initiated a series of processes that resulted in the death of cancer cells while leaving healthy tissues unharmed. This demonstrates the specificity and targeted effect enabled by the functionalized HMSNs. In addition, the dual catalytic capabilities of cerium resulted in antioxidant effects, which further improved the therapeutic results [94].

Estirado et al. (2024) investigated the application of platinum (Pt(II)) and palladium (Pd(II)) complexes in conjunction with a thiazoline derivative ligand. The covalent functionalization of these compounds onto mesoporous silica resulted in a notable enhancement in both selectivity and accumulation, specifically in HeLa cancer cells. The thiazoline derivative enabled selective ligand–receptor interactions, ensuring the preferential uptake of the antioxidant and therapeutic compounds by cancer cells. This enhanced the efficacy and reduced off-target effects [87].

These findings elucidate the crucial ways by which functionalized mesoporous silica might accomplish targeted distribution and enhance the selectivity of antioxidant drugs.

Functional groups that are responsive to certain stimuli, such as pH, reactive oxygen species (ROS), and redox conditions, facilitate the controlled release of medications in the desired environment. This ensures that the therapeutic agents exert their effects precisely at the intended site. Surface alterations using certain ligands, such as thiazoline derivatives, might enhance the attraction and absorption of drug-loaded nanoparticles by target cells, such as cancer cells, through a process called receptor-mediated endocytosis. By functionalizing mesoporous silica with certain moieties that have a preferential interaction with disease markers or microenvironments such as acidic tumor pH levels or high ROS levels, the antioxidant medicines can be selectively administered to the diseased cells. Functionalized mesoporous silica decreases the exposure of healthy tissues to medications by specifically delivering them to the targeted cells or their surroundings. This minimizes side effects and improves the overall safety of the treatment.

To summarize, functionalized mesoporous silica provides a robust framework for the precise administration and enhanced specificity of antioxidant medications. The customized surface chemistry and ability to respond to stimuli allow for accurate targeting and controlled release, greatly improving the therapeutic benefits of antioxidants. Future research should focus on investigating innovative methods of functionalization and their ability to specifically target different disease states, hence enhancing the efficiency and safety of antioxidant treatments.

#### 5.1.4. Improve Biocompatibility and Safety Profile

Functionalized mesoporous silica greatly improves the biocompatibility of antioxidant drugs in comparison to non-functionalized mesoporous silica. This improvement is mainly linked to the presence of diverse functional groups and surface modifications that interact positively with biological systems, resulting in decreased toxicity and enhanced cellular connections.

Functionalized mesoporous silica enhances biocompatibility by reducing cytotoxicity, improving hemocompatibility, and maintaining stability in biological environments. These enhancements directly increase the therapeutic efficacy of antioxidant drugs and reduce potential adverse reactions, rendering them more secure and efficient for clinical application.

Rasool et al. (2022) created a silica–ceria nanocomposite (FSC) that has been modified to be effective in the treatment of biofilms. The FSC incorporated the large surface area of mesoporous silica nanoparticles (MSNs) in conjunction with the inherent antibacterial properties of cerium oxide. Although the FSC is a positively charged nanomaterial, it showed great compatibility with human blood and effectively promoted the healing of wounds in a mouse fibroblast cell line within 24 h. Incorporating cerium oxide into the nanocomposite not only provided antibacterial capabilities, but also improved the overall biocompatibility of the material. These findings indicate that the addition of biocompatible substances such as cerium oxide to mesoporous silica can greatly decrease the harmful effects on cells and stimulate the process of healing, hence improving the effectiveness of antioxidant medications [100].

Xu et al. (2023) enclosed betanin into nanoparticles made of chitosan–sodium tripolyphosphate-coated quaternary ammonium-functionalized mesoporous silica (CS@QAMSNP). The composite that was obtained showed a cell viability of over 80%, which indicates a high level of biocompatibility. The chitosan coating created a surface that is compatible with living organisms, while the quaternary ammonium functionalization guaranteed the stability, and prevented the clumping together, of particles. The incorporation of this combination enhanced the biocompatibility of the enclosed betanin, thereby augmenting its capacity to hinder the production of advanced glycation end-products (AGEs) without eliciting any detrimental effects on the cells [28].

Brezoiu et al. (2020) evaluated the biocompatibility of Mamaia (MM) grape pomace polyphenolic extract when applied to both untreated and modified MCM-41 mesoporous silica. The materials loaded with extract exhibited excellent cytocompatibility at the concentrations evaluated, suggesting that their functionalization did not have any harmful consequences. The presence of functional groups on the surface of silica likely facilitated a more advantageous contact with cellular membranes, resulting in a decrease in toxicity and an improvement in the therapeutic effectiveness of the antioxidant polyphenolic extract [90].

Kerry et al. (2023) synthesized personalized mesoporous silica nanoparticles (MSiNPs) using the Stöber method and silane polymerization for the purpose of adsorbing Morin (MO). The results indicated that the MSiNPs adsorbed with MO were not poisonous and were compatible with living organisms, suggesting their potential as a successful carrier for drug delivery. The process of silane polymerization resulted in the formation of a surface that is stable and compatible with living organisms, hence minimizing any potential negative effects and improving the transportation and effectiveness of the antioxidant medication [102].

These studies emphasize various essential ways via which functionalized mesoporous silica can improve the compatibility of antioxidant medicines with living organisms, including applying biocompatible polymers, such as chitosan or bacterial-derived exopolysaccharides, to mesoporous silica, which creates a non-toxic surface that interacts positively with biological tissues. This reduces harmful effects on cells and improves the absorption of substances by cells. Introducing biocompatible elements like cerium and iron oxides through functionalization not only provides therapeutic benefits but also minimizes harmful interactions with biological systems, hence enhancing safety. Functional groups that have particular interactions with biological membranes can decrease non-specific interactions and limit cytotoxic effects, hence ensuring the safety of the drug delivery system for prolonged usage. Functionalization enhances the hemocompatibility of the drug delivery system, hence preventing any negative interactions with blood components and enabling its use for systemic administration. Functionalized mesoporous silica enhances the structural integrity of the medication in biological settings, hence maintaining its stability and efficacy during the entire delivery process.

Functionalized mesoporous silica provides a possible method to improve the compatibility of antioxidant medications with living organisms. By incorporating distinct functional groups and coatings, these materials have the ability to decrease toxicity, enhance cellular connections, and maintain stability in biological systems. As a result, they directly amplify the therapeutic benefits of antioxidants while minimizing any adverse effects. Further investigation should prioritize the exploration of innovative methods for functionalizing antioxidant medications and assessing their effects on different drugs in order to enhance their clinical utility.

#### 5.1.5. Improve Penetration

Functionalized mesoporous silica significantly enhances the penetration of antioxidant drugs in comparison to non-functionalized mesoporous silica. This improvement is mainly due to the integration of functional groups and the modification of surfaces that promote interaction with biological barriers, such as the blood–brain barrier (BBB), and enhance the absorption by cells.

Functionalized mesoporous silica enhances drug penetration by several mechanisms, such as targeting specific transport channels, boosting drug solubility, and improving cellular internalization. These enhancements directly optimize the therapeutic efficacy of antioxidant medications by improving their targeted tissue delivery.

Zou et al. (2022) developed hollow mesoporous manganese-doped silica nanoparticles (LHMMSN) that were functionalized with lactoferrin. These nanoparticles were designed to transport the insoluble medicine resveratrol (RES). The addition of lactoferrin was crucial in facilitating the penetration of these nanoparticles through the blood–brain barrier (BBB) and their specific targeting of inflammatory regions. Lactoferrin is recognized for its capacity to attach to lactoferrin receptors on the blood–brain barrier (BBB), thereby aiding in the transportation of nanoparticles into the brain. Upon entering the brain, LHMMSN-RES enhanced the functioning of antioxidant enzymes such as superoxide dismutase (SOD) and glutathione peroxidase (GSH-Px), decreased the presence of oxidative stress indicators, and regulated inflammatory and apoptotic processes. Its focused infiltration and consequent antioxidant efficacy illustrate the ability of functionalized mesoporous silica to enhance the transportation of drugs to inaccessible regions, such as the brain, thus amplifying the curative impact of antioxidants [95].

The enhanced penetration of antioxidant medications with the use of functionalized mesoporous silica can be attributed to multiple fundamental mechanisms. By functionalizing nanoparticles with ligands such as lactoferrin, they are able to utilize receptor-mediated endocytosis. This is a process in which nanoparticles connect to certain receptors on cell surfaces, including the blood–brain barrier (BBB), to facilitate their transportation across biological barriers. The presence of functional groups on the surface of mesoporous silica can augment the solubility and stability of hydrophobic medicines such as resveratrol, hence enhancing their bioavailability and capacity to penetrate target tissues. Functionalized mesoporous silica nanoparticles have a small particle size of approximately 140 nm and have an optimal surface charge. These characteristics enable them to efficiently cross cellular membranes and biological barriers, hence improving the drug’s ability to reach its intended target. Surface changes can be engineered to specifically target particular tissues or cells, hence enhancing the precision and effectiveness of medication delivery. Functional groups that specifically target inflammatory areas can assure the precise accumulation of antioxidant medicines at these locations, therefore boosting their therapeutic effectiveness.

Functionalized mesoporous silica possesses the ability to enhance the delivery of antioxidant medicines, resulting in enhanced therapeutic effects. Functionalized mesoporous silica enhances the penetration of drugs, allowing higher concentrations of antioxidants to reach target tissues. This enables the antioxidants to exert their effects more efficiently, reducing oxidative stress and inflammation, and promoting cellular protection and recovery.

To summarize, functionalized mesoporous silica presents a viable method for improving the penetration and therapeutic effectiveness of antioxidant medications. The customized alterations to the surface and incorporation of certain functional groups enhance the interaction with biological barriers and target cells, hence optimizing the delivery of medications to their targeted locations of action. Future studies should continue to investigate innovative methods of modifying the properties of antioxidant medications and examine how these modifications affect their ability to penetrate tissues and their effectiveness in treating medical conditions. This will help to improve the practical use of these treatments in clinical settings.

## 6. Author Perspectives

**Figure 2:**
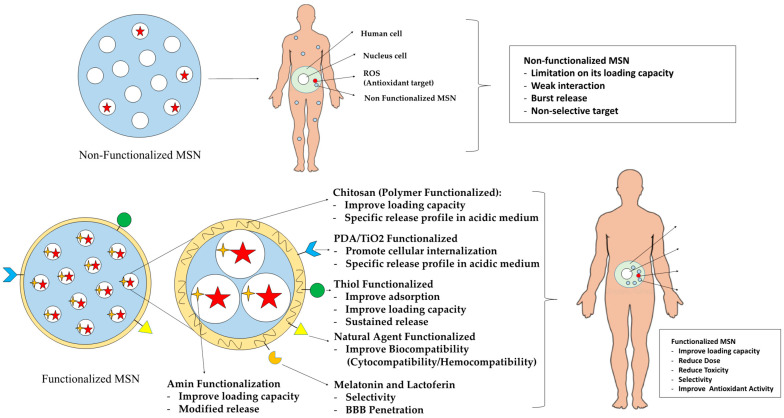
The speculated mechanism of functionalized mesoporous silica (FSM) in enhancing antioxidant activity.

Functionalized mesoporous silica is a versatile platform that greatly improves the delivery and effectiveness of antioxidant drugs through various linked-together mechanisms. These mechanisms include an increased loading capacity, controlled release behavior, targeted delivery systems, enhanced biocompatibility, and improved penetration. Each of these variables synergistically serves to optimize the therapeutic effects of antioxidant therapy (Figure 2).

The loading capacity of antioxidant medications is significantly increased by functionalizing mesoporous silica, since this process introduces certain functional groups that have a stronger interaction with drug molecules. Studies conducted by Kumari et al. (2021) and Xu et al. (2022) have shown that functional groups, such as thiols and quaternary ammonium compounds, have a substantial impact on the encapsulation effectiveness of quercetin and betanin, respectively [93,96]. These interactions are essential because they not only enhance the drug loading capacity of the silica but also enhance the stability and prevent early degradation, therefore assuring long-lasting therapeutic effectiveness.

Furthermore, the utilization of functionalized mesoporous silica allows for the precise and regulated discharge of antioxidant medications, a crucial factor in sustaining therapeutic concentrations for prolonged durations. Kumari et al. (2021) emphasized the use of pH-sensitive coatings or surface shifts, which enable targeted release in accordance with particular physiological conditions, such as acidic tumor microenvironments [93]. In addition, Purikova et al. (2022) have shown that stimuli-responsive systems such as ROS-sensitive switches can accurately deliver medications to specific locations and instances, hence improving the effectiveness of therapy while reducing unintended side effects [97].

Furthermore, functionalized mesoporous silica serves as a foundation for the precise administration of antioxidant medications, enhancing their specificity and effectiveness in affected tissues. Functional groups can be customized to preferentially interact with receptors or specific surroundings related to diseases, such as cancer cells or inflammatory tissues. This focused strategy not only amplifies the therapeutic impact but also mitigates harm to healthy tissues, therefore diminishing potential adverse reactions. The studies conducted by Fatemi et al. (2024) and Estirado et al. (2024) demonstrate how functionalized silica nanoparticles can be used to deliver medications directly to cancer cells using redox-responsive processes or particular ligand–receptor interactions [87,94].

Furthermore, the process of functionalizing mesoporous silica serves to enhance biocompatibility by diminishing cytotoxicity and augmenting the compatibility with biological systems. Rasool et al. (2022) and Xu et al. (2023) showed that functionalized silica-based nanocomposites had outstanding hemocompatibility and cell survival, which are crucial for systemic administration and clinical use. Applying biocompatible polymers as coatings or including physiologically inert materials such as cerium oxide can improve the safety and effectiveness of treatments [28,100].

Functionalized mesoporous silica improves the ability of antioxidant medications to enter specific tissues, such as the brain or inflamed areas, where they can effectively defend against oxidative stress. This is accomplished by utilizing mechanisms like receptor-mediated endocytosis, which is accelerated by certain surface ligands, or by enhancing the solubility and particle properties to facilitate cellular uptake. Zou et al. (2022) demonstrated the potential of functionalized silica nanoparticles to transport resveratrol through the blood–brain barrier, hence boosting its antioxidant benefits in neurological disorders [95].

Overall, the use of functionalized mesoporous silica shows great potential in improving the effectiveness and safety of antioxidant therapies. These materials provide an excellent basis for the creation of improved drug delivery systems by improving the loading capacity, permitting controlled release, facilitating targeted delivery, enhancing biocompatibility, and improving the penetration into target tissues. Future research should focus on developing new methods to enhance the functionality of antioxidant medications and investigate their potential use in a wider range of therapeutic applications. The goal is to improve clinical results and enhance patient care.

## 7. Conclusions and Future Perspective

Functionalized mesoporous silica offers a highly promising approach to enhancing the efficacy of antioxidant compounds. Functionalized mesoporous silica efficiently addresses critical challenges in antioxidant drug delivery by using its capacity to enhance loading, control release, provide targeted delivery, enhance biocompatibility, and facilitate penetration into certain tissues. These advancements not only improve the efficacy of treatment by augmenting the dosage of medication in the specific regions affected by the disease, but also minimize the probability of negative side effects by restricting interaction with healthy tissues.

The future of delivering antioxidant compounds using mesoporous silica shows great promise. Further investigation should prioritize the development of novel techniques to improve the characteristics of mesoporous silica by attaching a range of functional groups customized to meet specific therapeutic requirements to it. This may entail including stimuli-responsive components that allow for the precise regulation of antioxidant release in response to environmental signs, such as the pH, temperature, or levels of oxidative stress. In addition, investigating the utilization of mesoporous silica in conjunction with other nanomaterials may result in synergistic outcomes, hence enhancing the effectiveness of antioxidant delivery systems.

Furthermore, these enhanced mesoporous silica structures have significant potential for the application of a wider variety of antioxidant medicines. This has the potential to broaden their utilization beyond conventional applications, possibly targeting a range of diseases defined by oxidative stress, such as neurological disorders, cardiovascular diseases, and cancer. Furthermore, the effectiveness of customized medicine can be increased by developing mesoporous silica nanoparticles tailored to the specific requirements of each patient. This will result in optimized treatment plans and enhanced therapeutic results.

In conclusion, the continuous advancement in the process of adding specific functions to mesoporous silica shows great potential for the future of antioxidant treatments. Through further refinement of these materials and their application processes we can anticipate the development of more efficient and precise treatments that reduce side effects and optimize therapeutic benefits. This advancement has the capacity to greatly enhance the results for patients in many diseases situations.

## Figures and Tables

**Table 1:** Prevalent types of MSNs classified by their pore diameter.

Type	Pore Size	Structure	Properties	Limitations
MCM-41 (Mobil Crystalline Material-41)	2–6 nm	Ordered hexagonal arrangement of uniform pores	High surface area and well-defined pore structure; ideal for controlled drug delivery or separation processes involving small molecules	Small pore size limits capacity for larger molecules like therapeutic proteins or bioimaging agents
SBA-15 (Santa Barbara Amorphous-15)	5–30 nm	Ordered, large-pore, mesoporous silica with hexagonal arrangement	Larger pore size accommodates larger cargo molecules; suitable for drug delivery applications involving proteins, bioimaging agents, or gene delivery vectors	Amorphous nature can lead to slightly less uniform pore size distribution compared to MCM-41
MCM-48	2–30 nm	Ordered cubic arrangement of interconnected pores	Unique cubic pore structure provides a more intricate network for cargo loading and release; advantageous for controlled release applications or incorporating multiple functionalities within the MSN framework	Slightly more challenging to synthesize compared to hexagonal structures of MCM-41 and SBA-15
Other Mesoporous Silica Types	Various	Diverse pore sizes and structures	Examples include FDU-12 (lamellar pore structure for catalysis and controlled release) and TUD-1 (unique 3D network of interconnected pores for high surface area and efficient mass transport)	N/A

**Table 2:** Common Active Pharmaceutical Antioxidant Compounds, Adapted from data presented originally in Ref. [2].

No	Antioxidants	Antioxidant Acitivty	Solubility in Water
1	Alpha Mangostin	66.63 ± 34.65 µg/mL	2.03 × 10^−4^ mg in 1 L at 25 °C
2	α-tocopherol	0.059/0.01 mM	Insoluble in waterr
3	Ascorbic acid	8.9 ± 0.1 µg/mL	Soluble in water
4	Buthyl Hydroxy Toluene (BHT)	0.020 ± 0.001 µg/mL	Insoluble in water
5	Buthyl Hydroxy Anisole	0.035 ± 0.007 µg/mL	Insoluble in water
6	β-carotene	24.99 µg/mL	0.0006 g in 1 L at 25 °C
7	Curcumin	32.86 µM	3.12 mg in 1 L at 25 °C
8	Catechin	170.3 ± 2.0 µg/mL	0.45 mg in 1 mL at 25 °C
9	Quercetin	19.3 µ/mL	60 mg in 1 L
10	Lycopene	57.93 µ/mL	Insoluble in water
11	Lutein	35 µ/mL	Insoluble in water
12	Tertbutyl hydroquinone (TBHQ)	14.9 ± 0.81 mM	Practically insoluble in
13	Ferulic Acid	56.4 ± 4.6 µ/mL	0.78 g in 1 L
14	Myricitrin	32.7 ± 0.9 µ/mL	Practically insoluble in water
15	Ethyl Gallate	35.3 ± 4.0 µ/mL	Sparingly soluble in aqueous buffers
16	Resveratrol	0.49 ± 0.03 mM	3 mg in 100 mL
17	Rutin	2.77 ± 0.09 mM	12.5 mg in 100 mL
18	Kaempferol	0.82 ± 0.04 mM	0.18 g in 1 L
19	Myricetin	3.66 ± 0.30 mM	Very insoluble (<5 μg in 1 mL) in pure water
20	Isobavachalcone	250.8 µ/mL	Insoluble in water

**Table 3:** IC50 Value of Antioxidant Level.

IC50 µg/mL	Interpretation
<50	Very strong antioxidant activity
50–100	Strong antioxidant activity
101–150	Moderate antioxidant activity
150–250	Weak antioxidant activity
IC50 > 250	Very weak or negligible antioxidant activity

**Table 4:** Functionalized Mesoporous Silica Nanoparticle for Antioxidant Compound.

No	Drug Payload	Aims of Study	Functionalized Mesoporous Silica	Impact of Functionalized and Antioxidant Efficacy Result	References
1	Copper(II) complexes of cyclen and cyclam	Covalently immobilized copper(II) complexes in mesoporous silica aerogels for nanoenzyme applications with enhanced SOD activities. Exploring altered reactivities due to covalent immobilization and nanoporous confinement.	Aerogel polymer functionalized	Improve SOD activity	[86]
2	Platinum Pt(II) and Palladium Pd(II)	Improve the cytotoxic activity of metal complexes by supporting them on mesoporous silica nanoparticles (MSN) with a melatonin derivative (5 MT). Covalently functionalized systems for increased selectivity and reduced side effects.	Melatonin derivates (5-methoxytryptamine, 5 MT)	Improve selectivity Reduce dose Reduce potential side effect Improve antioxidant effect	[87]
3	Gallic acid, protocatechuic acid, chlorogenic acid, and 4-hydroxybenzoic acid	Investigate adsorption and desorption of various acids using commercially available mesoporous silica materials. Evaluate antioxidant potential using the Folin–Ciocalteu method.	Amine and Thiol	Improve loading capacity Improve antioxidant effect	[88]
4	N-(1,3-dimethyl)butyl-N′-phenyl-p-phenylenediamine	Synthesis of thiol-functionalized mesoporous silica nanorods loaded with antioxidant N-(1,3-dimethyl)butyl-N′-phenyl-p-phenylenediamine for anti-aging performance in styrene-butadiene rubber (SBR).	Thiol	Improve loading capacity Controlled release Prevent the bloom release Improve antioxidant effect	[89]
5	Mamaia grape pomace polyphenolic extract	Assess properties of Mamaia (MM) grape pomace polyphenolic extract loaded onto pristine and functionalized MCM-41 mesoporous silica. Evaluate the antioxidant effect on NIH3T3 cells.	Mercaptopropyl, propyl sulfonic acid, cyanoethyl and propionic acid	Improve release in PBS Improve cytocompatibility Improve RSA Improve antioxidant effect	[90]
6	Irganox	Incorporation of Irganox 1076 antioxidants into mesoporous MCM-41 silica for improving thermal stability and crystallization	Hybrid particles	Significantly improve Thermal stability Improve antioxidant effect	[91]
7	Orange Carotenoid Protein (OCP)	Immobilization of photoactive Orange Carotenoid Protein (OCP) on SBA-15 mesoporous silica nanoparticles for potential use as a photochromic material and antioxidant drug delivery.	Amine	Provide controlled release in appropriate condition Improve antioxidant effect	[92]
8	quercetin	Synthesis of chitosan-silica nanohybrid with quercetin for antibacterial and antioxidant properties.	Chitosan-Thiol	Improve antibacterial effect Improve antioxidant effect Has specific release in acidic medium	[93]
9	irinotecan	Introduction of multifunctional hollow mesoporous silica-based nanocarrier for targeted delivery of irinotecan to colorectal cancer cells. Incorporation of cerium and iron oxides for oxidative stress-mediated cytotoxicity.	Cerium, Iron oxide, and bacterial-derived exopolysaccharide (EPS) polymer	Greater cytotoxicity in cancer cells Selective delivery to cancerous tissue Induction of antioxidant effects.	[94]
10	Reserveratol	Design of lactoferrin-functionalized hollow mesoporous silica nanoparticles for delivery of insoluble drug resveratrol. Antioxidant stress, anti-inflammatory, and neuroprotective effects.	Lactoferin	Reduction in inflammation and protection of nerve cells Improved motor function recovery Improve penetration in BBB	[95]
11	Betanin	Encapsulation of betanin in chitosan-sodium tripolyphosphate coated quaternary ammonium-functionalized mesoporous silica nanoparticles for enhanced inhibition of AGEs formation.	Chitosan-tpp-amine	Higher inhibition rate of AGEs formation pH-dependent drug-releasing behavior Enhanced inhibition of methylglyoxal.	[96]
12	Cerium	Evaluation of ROS-responsive “nanogate” MSNs loaded with resveratrol for enhanced anticancer effects against lung and breast cancer cells.	methylthiopropyl	Improved anticancer effects against A549 and MDA-MB-231 cells Specific Release in ROS environment	[97]
13	Curcumin	Synthesis of inorganic-organic nanohybrid using amine-modified MCM-41 and chitosan succinate for controlled drug release, antibacterial activity, and antioxidant properties.	Chitosan-Amine	Controlled drug release with pH-dependent behavior Improve Antibacterial and antioxidant activities	[98]
14	Polyphenols-Reserveratol	Investigation of MSNs with ROS-responsive “nanogate” as drug delivery platforms for biomedical applications.	aminopropyl, isocyanate, phenyl, mercaptopropyl, and propionic acid	Enhanced anticancer effects against A549 and MDA-MB-231 cells. Specific release in acidic medium	[99]
15	Cerium oxide	Development of FSC as an antibiotic-free system for treating biofilms. Exhibits antioxidant activity, antibacterial effects, and potential for chronic wound healing applications.	Silica and ceria	Strong Inhibition against S. aureus and E. coli biofilms Hemocompatibility Good Potential for chronic wound healing. Improve safety Improve antioxidant effect	[100]
16	Atorvastatin	Synthesis of mesoporous SBA-16 silica nanocarrier functionalized with dopamine for in vivo delivery of atorvastatin.	silanizing agent, 3-amino-propyl-triethoxysilane, cyanuric chloride and dopamine	Improve antioxidant enzymes	[101]
17	Morin	Design a customized Mesoporous silica nanoparticle (MSiNPs) for Morin (MO) adsorption. Evaluate structural, thermal, and biological properties, with a focus on MO adsorption efficiency and biocompatibility.	Silane	Has Nontoxic and biocompatible Improve loading capacity. Reduce dose Improve biocompatibility Improve thermal stability Improve antioxidant efffect	[102]
18	Gold-Cerium	Develop thiolated, bioactive mesoporous silica nanoparticles (MSN-SH) for bone tissue regeneration. Investigate antioxidant properties, cell adhesion, osteogenic potential, and regenerative nature of MSN-SH.	Thiol	High antioxidant activity, neutralized ROS formed in cells, and provided protection against ROS-induced cell damage Induce cell-proliferative, Has a good osteogenic potential, and Induce regenerative nature	[103]
19	Polyphenolic flavonoids	Utilize engineered mesoporous silica nanoparticles (MSNPs) for nano harvesting of polyphenolic flavonoids from Solidago nemoralis hairy root cultures. Evaluate intracellular uptake, antioxidant activity, and pharmacological effects of the recovered solutes.	Yitanium dioxide (TiO_2_) and amines (NH_2_)	Increased antiradical activity Imrpove antioxidant effect	[104]
20	Ruthenium (ii)	Evaluate ruthenium(ii) complexes with pyridylimidazo[1,5-a]pyridine ligand on PDA-coated amino-functionalized mesoporous silica nanoparticles (AMSNs) for anticancer effects. Assess release profile, cellular internalization, and zebrafish embryotoxicity.	Amine	Enhanced cytotoxicity, Enhance anticancer potency, and cellular internalization Improve antioxidant effect	[105]
21	2-tert-butylhydroquinone (TBHQ)	Encapsulate 2-tert-butylhydroquinone (TBHQ) in mesoporous silica nanoparticles (MSNs) and amine-functionalized MSNs (AP-MSNs). Investigate release profiles, cytotoxicity, and antioxidant effects.	Amine	Has sustained release of TBHQ Increased antioxidant activity Lower cytotoxicity compared to free-TBHQ. Improve antioxidant effect	[106]
22	Tannic Acid	Investigate amino-functionalized tannic acid-templated mesoporous silica nanoparticles (TA-MS-NH2 NPs) for protection against iron-induced liver toxicity. Assess hepatotoxicity markers, oxidative stress, and acute iron toxicity.	Amine	Protection against iron-induced liver toxicity Improved hepatotoxicity markers and oxidative stress. Improve antioxidant effect	[107]
23	Silibinin	Develop hybrid mesoporous silica nanocomposites composed of polymerized alkyl methacrylate monomers for enhanced dissolution and antioxidant activity of Silibinin (SBN). Evaluate in-vitro dissolution and antioxidant activity.	alkyl methacrylate monomers	A remarkable increase in in-vitro dissolution and antioxidant activity of SBN Has specific release in acidic gastric medium.	[108]
24	Betanin	Encapsulate betanin in chitosan-sodium tripolyphosphate coated quaternary ammonium-functionalized mesoporous silica nanoparticles (CS@QAMSNPs). Evaluate morphology, loading capacity, antioxidant stability, and cell viability.	Chitosan-Tpp, and Amine functionalized	Improved stability of betanin Good safety High antioxidant activity.	[109]
25	Bioactive glass-ceramic (BGC) consisting of SiO_2_, CaO, Na_2_O, and P_2_O_5_	Functionalize bioactive glass-ceramic (BGC) particles with dopamine, glutamic acid, and cystamine dihydrochloride. Evaluate antibacterial, antioxidant, bioactivity, and dispersion stability properties for potential use in electrospinning and bioprinting.	Dopamine, glutamic acid, and cystamine dihydrochloride	Significant improve antibacterial, antioxidant, and bioactivity Good dispersion stability in dilute polymer solutions.	[110]
26	Cisplatin-Curcumin	Impregnate CuFe_2_O_4_/mesosilicalite and CuFe_2_O_4_/MCM-41 with cisplatin and curcumin for combinational drug therapy. Evaluate cytotoxic efficiency on various cell lines.	Magnetic spinel ferrite	Synergistic effect on reducing cell viability, triggering apoptotic signaling pathway Improve Antioxidant effect	[111]
27	Flavonoid-Vit C	Functionalize magnetized mesoporous silica material (Fe_3_O_4_NPs-UVM-7) with thiol and amine groups. Evaluate effects on enzymatic browning, physicochemical properties, bioactive compounds, and antioxidant capacity of cloudy apple juice.	Amine and Thiol	Reduced enzymatic browning; Maintained physicochemical properties and bioactive compounds Mitigated loss of total polyphenols and antioxidant capacity.	[112]
